# The Role of *M. leprae* Hsp65 Protein and Peptides in the Pathogenesis of Uveitis

**DOI:** 10.1155/2012/197648

**Published:** 2012-08-27

**Authors:** Alessandra Gonçalves Commodaro, Eliana Blini Marengo, Jean Pierre S. Peron, Wesley Brandao, Christina Arslanian, Robson Lopes Melo, Estevam J. Baldon, Rubens Belfort, Osvaldo Augusto Sant'Anna, Luiz Vicente Rizzo

**Affiliations:** ^1^Department of Ophthalmology, São Paulo Hospital, Federal University of São Paulo, R. Botucatu 820, 04023-062 São Paulo, SP, Brazil; ^2^Hospital Israelita Albert Einstein, Avenue Albert Einstein 627-701, 2 Subsolo Bloco A, 05651-901 São Paulo, SP, Brazil; ^3^Department of Immunology, University of São Paulo, R. Prof. Dr. Lineu Prestes 1730, 05508-900 São Paulo, SP, Brazil; ^4^Center for Applied Toxinology (CAT/CEPID), Butantan Institute, Avenue Vital Brazil 1500, 05503-900 São Paulo, SP, Brazil; ^5^Immunochemistry Laboratory, Butantan Institute, Avenue Vital Brazil 1500, 05503-900 São Paulo, SP, Brazil

## Abstract

Experimental autoimmune uveitis (EAU) is a well established model for immune-mediated organ-specific disease. Our group has recently shown that the *M. leprae* Hsp65 aggravated the uveitis in mice; in the present study, we evaluated the action of *M. leprae*  K^409^A mutant protein and the synthetic peptides Leader pep and K^409^A pep (covering amino acids residues 352–371 of WT and K^409^A proteins of *M. leprae* Hsp65, resp.) on the pathogenesis of EAU. Mice received the 161–180 IRBP peptide and *B. pertussis* toxin followed by the intraperitoneal inoculation of K^409^A protein or the Leader pep or K^409^A pep. The Leader pep aggravated the disease, but mice receiving the K^409^A pep did not develop the disease and presented an increase in IL-10 levels by spleen cells and a decrease in the percentage of CD4+ IFN-*γ*+ T cells. Moreover, animals receiving the Leader pep presented the highest scores of the disease associated with increase percentage of CD4+ IFN-*γ*+ T cells. These results would contribute to understanding of the pathogenesis of EAU and support the concept that immune responses to Hsp are of potential importance in exacerbating, perpetuating, or even controlling organ-restricted autoimmune diseases, and it is discussed the irreversibility of autoimmune syndromes.

## 1. Introduction 

Experimental autoimmune uveitis (EAU) is an organ-specific, T-cell-mediated disease that targets the posterior pole of the eye [1] and is characterized by granuloma formation in the neural retina, destruction of photoreceptor cells, and blindness [1, 2].

The Hsp60 family comprises molecules that are immunodominant in several infectious processes [3]. The *Mycobacterium leprae* Hsp65 is part of this family-denominated chaperonins and one of the major immuno-reactive proteins in mycobacteria [3]. It is noteworthy that this protein shares approximately 50% amino acid identity with its mammalian homologue, suggesting their participation in the autoimmune diseases by molecular mimicry mechanism between self and exogenous Hsp molecules [4, 5]. The role of the Hsp65 has been intensively investigated in the pathogenesis of arthritis and diabetes [6, 7], murine systemic lupus erythematosus [8], ocular manifestations of Behcet's disease, and acute anterior uveitis [9, 10]. High anti-Hsp65 antibodies titers were correlated to the retinopathy in type I diabetes patients [11] and with the development and progression of atherosclerosis lesions [12, 13].

Moreover, in animals with acute EAE, lesions were accentuated by increased expression of Hsp60, primarily by infiltrating cells. In chronic EAE, Hsp60 was found predominantly on CNS components, particularly oligodendrocytes and astrocytes, showing the participation of Hsp in CNS [14]. In addition, the 180–188 mycobacterial Hsp65 epitope, which is cross-reactive with a self-antigen in joint cartilage, is able to initiate and induce resistance to subsequent attempts to produce the adjuvant arthritis [6, 15].

Our group previously exposed that the passive administration of wild-type* M. leprae* Hsp65 interfered with endogenous equilibrium by enhancing the entropy of the immunobiological system, as expressed by the early death of the (NZB×NZW)F_1_ experimental lupus mice [8] and aggravation of the ocular disease in experimental autoimmune Uveitis [16]. Following these studies, the biological effects and the primary sequence of *M. leprae* Hsp65 Leader pep and K^409^A pep synthetic peptides, which cover residues 352–371, were shown [17].

In this context, in the present study, we evaluated the effects of the K^409^A mutant Hsp65 protein and the Leader pep and K^409^A pep peptides administration in the pathogenesis of EAU. We showed that K^409^A and Leader pep were able to aggravate the disease. On the other hand, mice injected with K^409^A pep did not develop the disease when compared to controls, K^409^A, and Leader pep.

## 2. Methods

### 2.1. Animals

Six- to eight-week-old B10.RIII mice were obtained from the animal facilities at the University of São Paulo, Brazil. All animals were housed under specific pathogen-free conditions and handled under ethical conditions. The Animal Care Committee of the Institute of Biomedical Sciences at the University of São Paulo approved all the procedures used in this study.

### 2.2. Expression of the Recombinant K^409^A Mutant *M. leprae* Hsp65 in *Escherichia coli* and Purification

Expression and purification of the recombinant K^409^A mutant *M. leprae *Hsp65 was done as described in [8].

### 2.3. Peptide Synthesis

Leader pep (ENSDSDYDREKLQERLAKLA) of *M. leprae* Hsp65 and K^409^A pep (ENSDSDYDREALQERLAKLA) of the mutated form K^409^A, both covering residues 352–371, were synthesized using the Fmoc (N-(9-fluorenyl)methoxycarbonyl) procedure [18] in a Shimadzu PSSM8 peptide synthesizer (Shimadzu, Tokyo, Japan). The Fmoc-amino acids were purchased from Novabiochem (Nottingham, UK). The synthetic peptides were purified by preparative reversed-phase chromatography (reversed-phase HPLC), and the purity and identity of the peptides were confirmed by matrix-assisted laser desorption ionization time-of-flight (MALDI-TOF) mass spectrometry on Ettan MALDI-TOF/Pro instrument (Amersham Biosciences, Buckinghamshire, UK) and by analytical reversed-phase high profile liquid chromatography (HPLC) (Shimadzu Inc., Tokyo, Japan).

### 2.4. Induction of EAU and *In Vivo* Treatment with Hsp65 Molecules

Mice [*n* = 4–6/group] were immunized subcutaneously (s.c.) at the base of the tail with 40 *μ*g of interphotoreceptor retinoid binding (IRBP) 161–180 peptide emulsified in 0.2 mL of complete Freund's adjuvant (CFA) (v/v). At the same time, mice were injected intraperitoneally (i.p.) with 0.5 *μ*g of *Bordetella pertussis* toxin (PTX) in 0.1 mL as an additional adjuvant and followed by single-dose inoculation of 2.5 *μ*g of K^409^A, Leader pep, or K^409^A pep i.p.

### 2.5. Histopathology EAU

Eyes were collected and prepared for histopathological evaluation at the end of each experiment (day 21 after immunization). The eyes were immersed for 1 h in phosphate-buffered glutaraldehyde 4%, transferred into phosphate-buffered formaldehyde 10% for 24 h, and replaced with ethanol 70% until processing. Fixed and dehydrated tissue was embedded in paraffin wax, and 4–6 *μ*m sections were cut through the papillary-optic nerve plane. Sections were stained by hematoxylin and eosin. Presence or absence of disease was evaluated in a double-blinded fashion by examining six sections cut at different levels for each eye. Severity of EAU was scored on a scale of 0 (no disease) to 4 (maximum disease) in half-point increments, according to a semiquantitative system described previously [1], according to lesion type, size, and number. In brief, the minimal criterion to score an eye as positive by histopathology was inflammatory cell infiltration of the ciliary body, choroids, or retina (EAU grade 0.5). Progressively higher grades were assigned for the presence of discrete lesions in the tissue such as vasculitis, granuloma formation, retinal folding and/or detachment, and photoreceptor damage [1]. 

### 2.6. Determination of Cytokine Production

Spleen cells harvested 21 days after immunization were cultured in 24-well plates (10^6^ cells/well) and stimulated with 30 *μ*g/mL IRBP. Supernatants were collected for cytokine analysis after 72 h and stored at −80°C until assayed. The level of IL-10 was assessed by ELISA using kit from BD/ Pharmingen (La Jolla, CA), and the level of IL-17 was assessed by ELISA using kit from eBioscience (San Diego, CA). All kits were used according to the manufacturer's instructions.

### 2.7. Intracellular Cytokine Staining and FACS Analysis

For detection of intracellular expression of IFN-*γ*, cells from draining lymph nodes and spleens collected at day 21 after immunization were labeled with anti-CD4 monoclonal antibody, fixed and permeabilized, stained intracellularly, and analyzed by flow cytometry evaluating the CD4 and IFN-*γ* expression. Cells were left unstimulated or stimulated overnight with IRBP (30 *μ*g/mL) in the presence of either Golgiplug or Golgistop at the recommended concentrations (BD Pharmingen). All samples were acquired on a FACSCalibur (BD Biosciences), and data were analyzed with FlowJo software (TreeStar).

### 2.8. Statistical Analysis

Data are expressed as mean ± SD. Statistical analyses were performed using GraphPad Prism version 5.00 for Windows (GraphPad Software, San Diego California, USA). Parametric Student's *t-*test was employed, and *P* values <0.05 were considered significant.

## 3. Results

### 3.1. K^409^A, Leader Pep, and K^409^A Pep in the Disease Development in Mice

**Figure 1:**
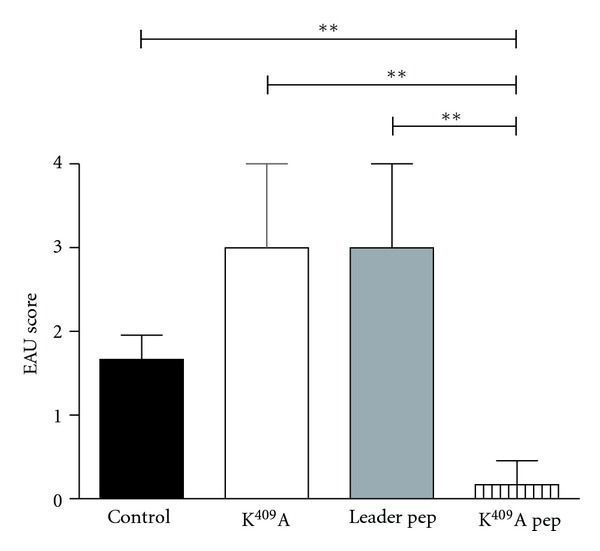
Administration of K^409^A protein and Leader pep increased EAU scores, and injection with K^409^A pep decreased EAU scores. B10.RIII mice were immunized with 40 *μ*g 161–180 IRBP peptide on day 0 and followed by a single injection with 2.5 *μ*g of K^409^A, Leader pep, or K^409^A pep (i.p.). Eyes were collected for histopathology 21 days after immunization. EAU scores were assigned by histopathologic examination of the eyes on a scale from 0 to 4 according to the extent of inflammation and tissue damage. **P < 0.01: control *versus* K^409^A pep; K^409^A *versus* K^409^A pep; Leader pep *versus* K^409^A pep. Disease incidence: control group: 75% of animals presented grade 1.5; K^409^A: 75% of animals expressed grade 3; Leader pep: 75% of animals presented grade 3; K^409^A pep: 75% of animals expressed no disease.

**Figure 2:**
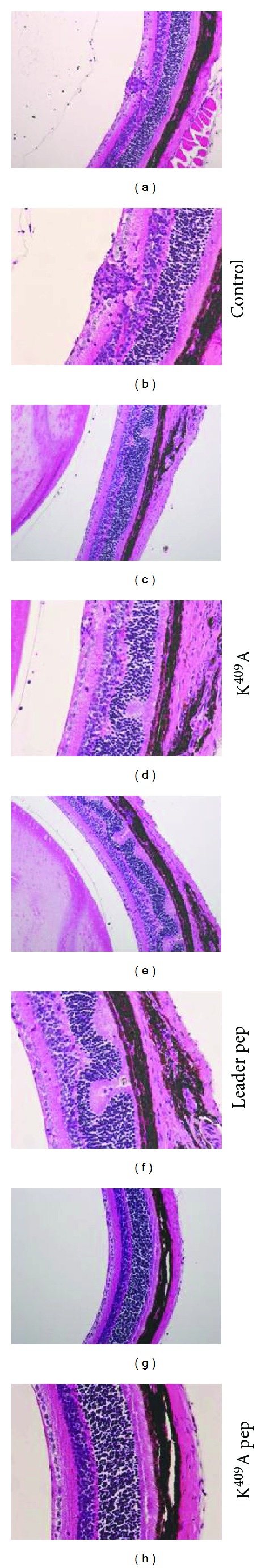
Histopathologic features representative of the EAU scores. Mice control shows ocular lesions characterized by cells infiltrating the vitreous and vasculitis (a) and (b); in mice injected with K^409^A, it is possible to observe inflammatory cells in the vitreous and retinal folds (c) and (d); mice injected with Leader pep show cells in the vitreous and retinal folds (e) and (f); mice injected with K^409^A pep exhibit a normal retinal architecture corresponding to nonimmunized naïve mice (g) and (h). Representative photographs (hematoxylin-eosin staining). Original magnifications: 100× (left); 400× (right).

The animals were immunized and injected with K^409^A, Leader pep, or K^409^A pep. We observed that K^409^A and Leader pep aggravated the development of the disease when compared with K^409^A pep and control group (Figure 1). On the other hand, animals injected with K^409^A pep did not develop the disease (Figure 1). The histopathologic examination shows vasculitis and presence of inflammatory cells in the vitreous in mice with EAU on day 21 (Figures 2(a) and 2(b), controls). In animals injected with K^409^A (Figures 2(c) and 2(d)) and Leader pep (Figures 2(e) and 2(f)), it is possible to observe the presence of inflammatory cells in the vitreous and retinal folds. On the other hand, in animals injected with K^409^A pep we can observe a normal organization of the retina (Figures 2(g) and 2(h)). 

### 3.2. Decrease of T CD4+ IFN-*γ*+ Cell Population in Lymph Nodes and Spleens of Mice Injected with K^409^A Pep

**Figure 3:**
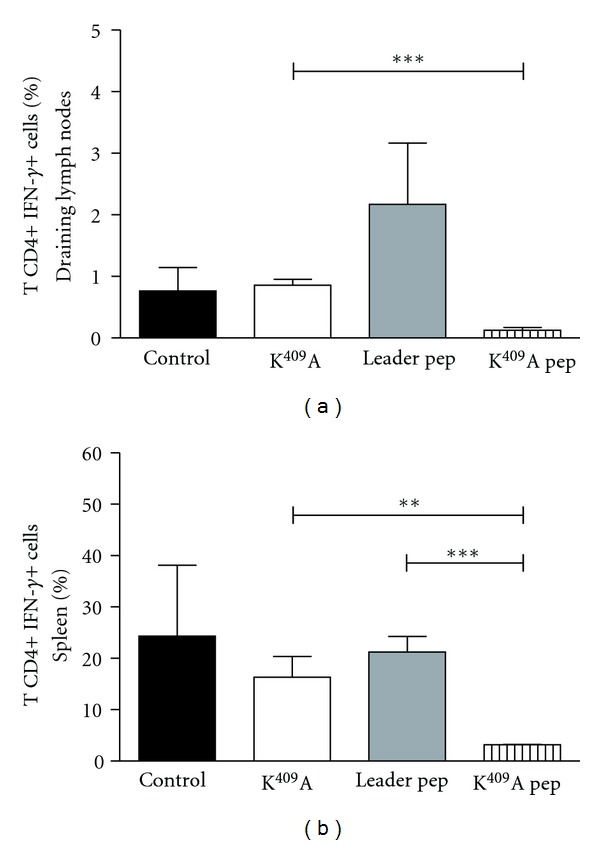
Expansion of CD4+IFN-*γ*+ T cells in lymph nodes (a) and spleen (b) after K^409^A, Leader pep, and K^409^A pep inoculation. Cells from draining lymph nodes and spleen collected at day 21 after immunization were labeled with anti-CD4 monoclonal antibody, fixed and permeabilized, stained intracellularly, and analyzed by flow cytometry evaluating the CD4, IFN- expression. Results are expressed in mean and SD. ***P < 0.001: K^409^A *versus* K^409^A pep (A); **P < 0.01: K^409^A *versus* K^409^A pep (B); ***P < 0.001: Leader pep *versus* K^409^A pep (B).

It is known that autoreactive Th1 cells mediate EAU, and thus its induction correlates with the production of IFN-*γ* by T cells. In this work, we observe a significant increase in the frequency of CD4+IFN-*γ*+ T cells in lymph nodes (Figure 3(a)) of animals injected with Leader pep when compared to those of K^409^A, K^409^A pep, and control groups. Also, there is a decrease of CD4+IFN-*γ*+ T cells in lymph nodes and spleen of the K^409^A pep mice group compared to the other experimental groups (Figures 3(a) and 3(b)). These results corroborate with our score results where we showed that mice injected with K^409^A pep presented lower scores of the disease when compared to mice injected with K^409^A, Leader pep, and controls.

### 3.3. Analysis of IL-10 and IL-17 Levels in Spleen

**Figure 4:**
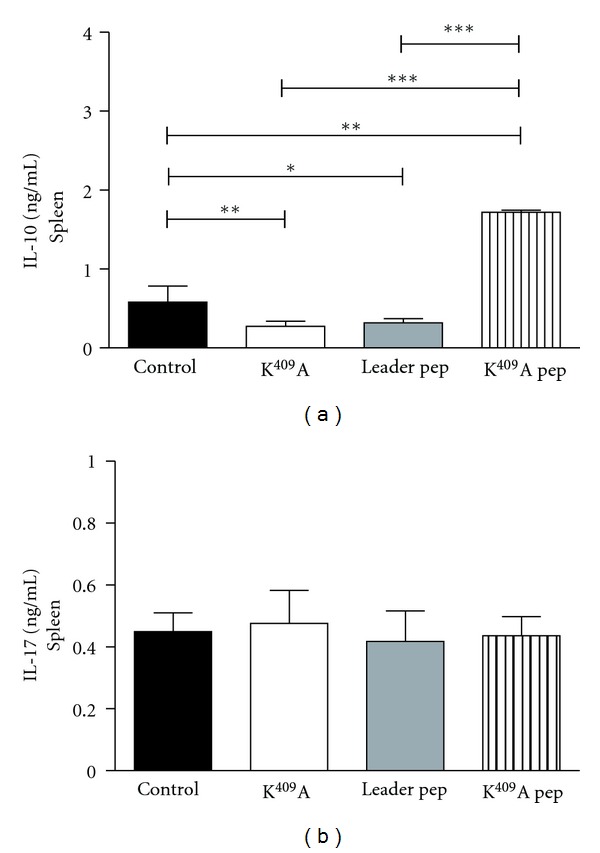
Analyses of IL-10 and IL-17 levels. Spleen cells from K^409^A, Leader pep, and K^409^A pep inoculated, or control mice were harvested at day 21 and stimulated *in vitro* with 30 mg/mL IRBP. After 72 hours, IL-10 (a) and IL-17 (b) levels were determined by ELISA. Results are expressed as mean ± SD. **P < 0.01: control *versus* K^409^A (A); *P < 0.05: control *versus* Leader pep (A); **P < 0.01: control *versus* K^409^A pep (A); ***P < 0.001: K^409^A *versus* K^409^A pep (A); ***P < 0.001: Leader pep *versus* K^409^A pep (A).

The immunosuppressive cytokine IL-10 regulates EAU susceptibility and may be a factor in genetic resistance to EAU [19]. In this work, we showed an increase in IL-10 levels by mice injected with K^409^A pep when compared to other groups. This result corroborates the EAU score presented by K^409^A pep mice, in which the animals did not develop the disease (Figure 4(a)). In regard of IL-17 production, it was observed that spleen cells of animals injected with K^409^A, Leader pep, and K^409^A pep produced the same amount of cytokines as the control animals (Figure 4(b)).

## 4. Discussion 

Recently, we showed that EAU scores are increased in IRBP-immunized animals and inoculated with recombinant wild-type *M. leprae* Hsp65. Interestingly, this was associated with a higher expansion of CD4+IFN-*γ*+ (Th1) and CD4+IL-17+ T cells (Th17), and with higher levels of IFN-*γ* [16]. Moreover, Marengo and collaborators still demonstrated that WT Hsp65 inoculation accelerates death in mice which spontaneously develop the Systemic Lupus Erythematosus (SLE) [8].

In the present work, we showed higher EAU scores in animals injected with K^409^A and Leader pep. However, animals injected with K^409^A pep did not develop the disease. Histopathological analysis showed that animals injected with K^409^A pep did not show lesions of retina when compared to controls, otherwise K^409^A and Leader pep presented retinal folds, vasculitis, and inflammatory infiltrating cells in the vitreous. 

To evaluate the effect of K^409^A, Leader pep, and K^409^A pep in the periphery, it was evaluated the CD4+ T cells producing IFN-*γ* in spleen and lymph nodes of mice induced to EAU. In this model, the increase in CD4+ IFN-*γ* + T-cell population in lymph nodes and spleens was associated with the increased scores observed in the K^409^A, Leader pep, and control groups. Friedland and collaborators demonstrated that the secretion of proinflammatory cytokines from monocytes activated by mycobacterial 65 kDa Hsp may be important in the host immune response and in the development of antigen-specific T-cell-mediated immunity [20].

Th17 cells were recently described as crucial for the development of EAU [21–25]; however, it was not observed differences in IL-17 levels by splenocytes at day 21 after-immunization between the experimental groups. Thus, we may assume that, at this phase, the Th17 cells are in the eye. On the other hand, different from that we observed in the present work, it was previously shown an expansion of CD4^+^IL-17^+^ T cells in mice injected with rHsp65 [16]. 

It is known that the immunosuppressive cytokine IL-10 regulates EAU susceptibility and may be a factor in genetic resistance to EAU [19]; the results showed that spleen cells from animals injected with K^409^A pep produced higher levels of IL-10. The increase of IL-10 levels can be associated with the absence of the disease, as IL-10 is a potent suppressive cytokine. Moreover, as we did not evaluate Tregs, it is also possible that higher levels of IL-10 observed in Leader pep mice group may be derived from this population such as Tr1 cells or IL-10 secreting Foxp3^+^ cells. 

In 2006, our group showed that rGal-1 administration was able to decrease the disease in mice with EAU, and this fact was associated with increased levels of IL-10 and decreased levels of IFN-*γ* [26]. A study with experimental arthritis showed that antibodies against the Hsp molecule suppress inflammation by inhibiting the proinflammatory effect of the Hsp on the innate immune system. The increase in IL-10 secretion in the inflammatory site can skew the local cytokine profile from an inflammatory to an anti-inflammatory response and thus explain the mechanism of protection against inflammation by these antibodies [27]. 

The opposite effects of the K^409^A protein and their correspondent peptide which covers the 352–371 residues, the K^409^A pep, in EAU score were unexpected. In (NZB×NZW)F_1_ mice, which present the H-2^d/z^ alleles, the inoculation of the Leader pep and K^409^A pep shows resembled effects to their respectively proteins in the survival time, showing a amplified effect [17]. Here, in the EAU susceptible B10.RIII mice, the H-2^r^ allele can explain at least in part their divergent effect. We can speculate that an important participation of the genetic background and immunobiological factors occurs. EAU is a organ-specific disease mediated by T-cells commitment, and SLE is a systemic disease characterized mainly by humoral self-response. Furthermore, differences at the H-2 loci in B10.RIII and (NZB×NZW)F_1_ mice suggest different or selective antigen-binding core and presentation to the immune system. Also, we can consider that the short length of the K^409^A pep facilitates its antigen processing of these cells, being more effective than the K^409^A protein. Some theoretical experiments based on molecules interaction by structural modeling studies can clarify the MHC molecules peptide binding to better comprehend the *in vivo* findings observed to the mutant forms.

**Figure 5:**
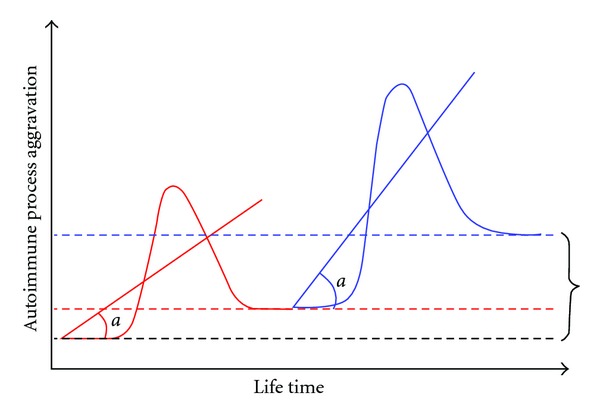
Autoimmune disease aggravation: *Y*: the individual state of an irreversible immune responsiveness; *a*: disease degree; ▲_*i*_: entropy production, where *i* represents the number of autoimmune episodes being a time-dependent discrete variable.

Figure 5 is based on the linear equation *y* = *a* +**▲**_*i*_: this theoretical model presupposes that in anautoimmune process, it must be considered the entropy production [▲] during the disease progression where the slope a is the intensity and degree of the process aggravation; ▲, although variable to each individual, is a constancy for everyone, increasing at all illness episode [_*i*_]. It was formulated the principle that *the immunological history of an individual is unique and irreversible*, being cumulative in an autoimmune process, staying progressively far from the physiological equilibrium [8]; thus, as for other autoimmunities syndromes, the uveitis is not reversible in the sense that the original immune state before the beginning of the disease cannot be reacquired. The general physiological conditions could be disrupted by similar homologous proteins, such as the *M. leprae* Hsp that, along the life time, proceeds performing a pathological role. At each new episode, the entropy production is higher, and the disease progression could only be controlled or delayed.

In conclusion, K^409^A and Leader pep were able to aggravate EAU development when administrated at the same time with the immunization. Moreover, mice injected with Leader pep presented high scores of the disease associated with increase percentage of CD4+IFN-*γ*+ T cells. However, K^409^A pep was able to inhibit the development of EAU, and this fact can be associated with an increase observed in IL-10 levels by spleen cells and with a decrease in the percentage of CD4+IFN-*γ*+ T cells. The results of this study would contribute significantly to understanding of the role of the Hsp molecules in the pathogenesis of ocular autoimmune diseases. They also support the concept that immune responses to Hsp have potential importance in exacerbating and perpetuating organ-restricted autoimmune diseases.
